# Evidence for the Primary Role of Phytoplankton on Nitrogen Cycle in a Subtropical Reservoir: Reflected by the Stable Isotope Ratios of Particulate Nitrogen and Total Dissolved Nitrogen

**DOI:** 10.3389/fmicb.2019.02202

**Published:** 2019-09-25

**Authors:** Yangyang Cai, Yingjie Cao, Changyuan Tang

**Affiliations:** ^1^School of Environmental Sciences and Engineering, Sun Yat-sen University, Guangzhou, China; ^2^Guangdong Provincial Key Laboratory of Environmental Pollution Control and Remediation Technology, Sun Yat-sen University, Guangzhou, China; ^3^School of Geography and Planning, Sun Yat-sen University, Guangzhou, China

**Keywords:** phytoplankton, nitrogen stable isotope, subtropical reservoirs, nitrogen cycle, microbial ecology

## Abstract

Knowledge about the primary factor controlling stable isotope ratios of particulate nitrogen (δ^15^N_PN_) and total dissolved nitrogen (δ^15^N_TDN_) in a subtropical reservoir can improve the understanding of regional and global nitrogen cycles. Taking Lianhe Reservoir as a representative subtropical reservoir, we studied the spatial and temporal distributions of δ^15^N_PN_ andδ^15^N_TDN_ and their relationships with the surrounding physicochemical factors and phytoplankton. The results showed that variations in δ^15^N_PN_ and δ^15^N_TDN_ followed seasonal thermal cycles. The values of δ^15^N_TDN_ were inversely proportional to those of δ^15^N_PN_. PCA showed that phytoplankton cell density and pH were the primary drivers of the variation of δ^15^N_PN_ (45.2%). The primary factors influencing δ^15^N_TDN_ were Chl *a* and phytoplankton cell density, which both indicated phytoplankton biomass. We also determined that the dominant species was *Microcystis densa* during the thermal stratification period and *Staurodesmus aristiferus* during the mixing period. Laboratory experiments showed that δ^15^N_PN_ values in both *M. densa* (from 19.5 to 14.6‰) and *S. aristiferus* (from 19.4 to 16.0 ‰) media decreased significantly as the algal cells grew. Furthermore, the δ^15^N_TDN_ values increased from 4.9 to 7.9‰ and from 4.7 to 6.9‰ in *M. densa* and *S. aristiferus* media, respectively, when the δ^15^N_PN_ values decreased. These experimental results were consistent with field investigation results and indicated that variations in δ^15^N_PN_ and δ^15^N_TDN_ were mainly controlled by phytoplankton cell density, especially the cell density of the dominant species, in both the thermal stratification and mixing periods. The results also suggested that cell density, not phytoplankton species, was the key factor regulating the distribution of nitrogen stable isotopes. These results together indicated that phytoplankton cell density is the primary factor in the regulation of nitrogen stable isotope composition and that its influence is greater than that of other physical and chemical factors. This study provided detailed information supporting the primary role of phytoplankton in the nitrogen geochemical cycle and improved the understanding of biochemical processes in natural subtropical reservoirs.

## Introduction

Nitrogen pollution has become a serious environmental problem in aquatic ecosystems worldwide (Smith and Schindler, 2009). Large amounts of industrial, agricultural, and urban nitrogen pollutants are discharged into rivers, reducing water quality (Bu et al., 2011; Chen et al., 2019). These increased nitrogen loadings in rivers flow into lakes and reservoirs, causing algal biomass development, and even algal blooms (Dodds et al., 2009; Gao et al., 2018). Sewage pollutants heavy in nitrogen in wastewater treatment plants are removed by denitrifying bacteria (Zhang et al., 2019). The microbial community in aquatic ecosystems is closely related to nitrogen migration and transformation.

The nitrogen stable isotope ratio (δ^15^N) is an effective tool for studying the nitrogen (N) cycle in aquatic systems. δ^15^N is also used as a tracer for detecting the distribution of pollutants and the amplification of biological effects on pollutants. δ^15^N can produce an integrative picture of chemical and biochemical N transformations (Waser et al., 1998; Granger et al., 2004). Therefore, the N stable isotope ratios of particulate nitrogen (δ^15^N_PN_) and total dissolved nitrogen (δ^15^N_TDN_) can be used to assess nitrogen sources and various nitrogen cycling processes, such as nitrification and denitrification, the uptake of nitrogen by phytoplankton and even food chains and biological webs (Lehmann et al., 2004; Hadas et al., 2009). There are different δ^15^N compositions in rivers, lakes, reservoirs and marine regions, because of characteristic living organisms and inanimate matter. Many studies have suggested that variations in δ^15^N_PN_ and δ^15^N_TDN_ are associated with thermal and hydrological characteristics, trophic states, nitrogen sources, N_2_ fixation, and phytoplankton abundance (Altabet, 2006; Gu et al., 2006, Gu, 2009; Gu and Schelske, 2010; Hou et al., 2013). However, the key factors that control variations in δ^15^N_PN_ and δ^15^N_TDN_ are not well understood. Clear and strong evidence for phytoplankton directly regulating the distribution of δ^15^N_PN_ and δ^15^N_TDN_ is still needed.

Phytoplankton, responsible for the primary productivity in aquatic systems, uptakes and assimilates nitrogen for photosynthesis and the biosynthesis of macromolecules, such as proteins, nucleic acids, and chlorophyll (Gao et al., 2018). These processes influence the nitrogen stable isotope composition in the water column (Needoba and Harrison, 2004). Sachs et al. (1999) showed that when nitrogen is sufficient in water column, algal cells preferentially absorb ^14^N, increasing the proportion of ^15^N_TDN_ and decreasing the proportion of ^15^N_PN_. However, when nitrogen is exhausted, algae preferentially absorb ^15^N, which weakens the fractionation of nitrogen stable isotopes. Nutrient supply, light intensity, phytoplankton species and nitrogen type are suggested to affect the ^15^N/^14^N uptake of phytoplankton (Waser et al., 1999; Doi et al., 2004). However, the effect of phytoplankton biomass on the absorption of ^15^N in a subtropical reservoir is still unclear.

In contrast to natural rivers and lakes, subtropical reservoirs are artificial aquatic systems with unique hydrological characteristics that experience the thermal stratification and mixing periods. Many subtropical reservoirs with similar hydrological and biochemical environments have experienced large-scale algal blooms, especially cyanobacterial blooms (Table 1). Lianhe Reservoir, located in a subtropical marine monsoon climate region, has also experienced a large outbreak of cyanobacteria. The temporal and spatial distributions of nitrogen in reservoirs are different from those before outbreaks because of the absorption and assimilation by large amounts of phytoplankton. The δ^15^N_PN_ and δ^15^N_TDN_ in the water column also change. Many studies have indicated the important role of phytoplankton in varying δ^15^N_PN_ and δ^15^N_TDN_ in rivers, lakes and marine areas (Sugimoto et al., 2014; Liu et al., 2017; Kharbush et al., 2019). However, the trends in δ^15^N_PN_ and δ^15^N_TDN_ in subtropical reservoirs might be less predictable than those listed above (Hou et al., 2013). Therefore, with Lianhe Reservoir as a classic example of a subtropical reservoir, it is necessary to study the relationship between phytoplankton cell density and seasonal variations in δ^15^N_PN_ and δ^15^N_TDN._ Our hypothesis is that phytoplankton cell density was the primary key factor controlling the temporal and spatial distributions of δ^15^N_PN_ and δ^15^N_TDN_ in subtropical reservoirs.

**TABLE 1:** Comparing the water temperature (WT, °C), dissolved oxygen (DO, mg l^–1^), NO_3_^–^-N (mg l^–1^), NH_4_^+^-N (mg l^–1^), total nitrogen (TN, mg l^–1^), total phosphorus (TP, mg l^–1^), and dominant algae during the thermal stratification period between the Lianhe Reservoir and other subtropical reservoirs.

**Reservoirs**	**WT**	**DO**	**NO_3_^–^-N**	**NH_4_^+^-N**	**TN**	**TP**	**Dominant algae**	**Location**	**References**
Lianhe	30.5	8.4	1.3	0.3	3.8	0.07	Cyanobacteria	China	–
Salto Grande	27	7.5	–	–	2.9	0.02	Cyanobacteria	Aragentina	O’Farrell et al., 2012
Caxias	30.4	7.6	0.4	0.1	1.41	0.01	Cyanobacteria	Brazil	Wojciechowski et al., 2017
Chopim	29.2	7.1	0.4	0.1	1.28	0.04	Cyanobacteria		
Tingxi	30	5	2	0.1	5.5	0.14	Cyanobacteria	China	Lv et al., 2014
Shidou	33.1	5.4	0.05	0.03	5.5	0.02	Cyanobacteria		Yang et al., 2012
Bantou	32.3	5.3	0.01	0.03	6.1	0.03	Cyanobacteria		
Fengtou	31.2	4.9	5.7	0.1	6.5	0.03	Cyanobacteria		

To comprehensively test our hypothesis, we carried out field surveys and laboratory experiments. The objective was to explore whether phytoplankton cell density plays a primary role in nitrogen migration and transformation that is more important than the roles of other physical and chemical factors. To fulfill our objective, we (1) investigated temporal and spatial variations in δ^15^N_PN_ and δ^15^N_TDN_, the phytoplankton community, and other physical, chemical and biological factors in Lianhe Reservoir; (2) analyzed the relationships between δ^15^N_PN_, δ^15^N_TDN_, phytoplankton and other physicochemical factors; and (3) used experiments to show that phytoplankton cell density was the key factor affecting variations in δ^15^N_PN_ and δ^15^N_TDN_. These results provide detailed information supporting the important role of phytoplankton in the nitrogen geochemical cycle and improve the understanding of the biochemical processes in subtropical reservoirs.

## Materials and Methods

### Study Area

**FIGURE 1:**
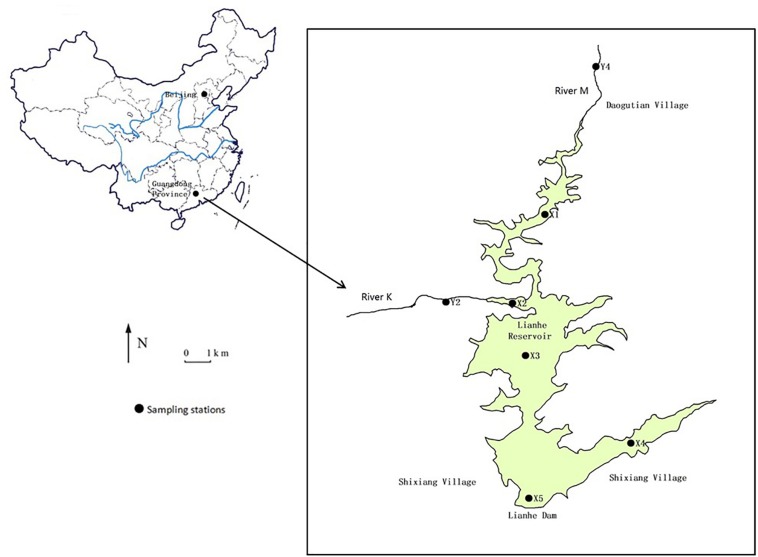
Illustration of Lianhe Reservoir showing the location of sampling stations in 2017.

Lianhe Reservoir (23°17′57.2′′N and 113°55′8.8′′E), located in Guangdong Province, South China, is a typical canyon-shaped reservoir in the subtropical marine monsoon region (Figure 1). The catchment area is 110.8 km^2^ with plenty of rainfall and heat in summer, and warmth and dryness in winter. The annual average rainfall and temperature is 1932.7 mm and 21.8°C, respectively. The dam was built in the southwest part of the study area in the 1970s, forming the reservoir with a surface area of 3.33 km^2^ and a storage capacity of 8.2 × 10^7^ m^3^. Lianhe Reservoir serves multiple purposes, including irrigation, hydroelectric power generation, flood control, and especially drinking water supply.

The hills surrounding the reservoir are covered with pine and spruce on the east side and eucalyptuses on the other sides. A small river K (stations Y2) flows into the reservoir. Another small river M (station Y4) flows into the reservoir from Daogutian village (Figure 1).

### Field Sampling and Methods

Water samples of Lianhe Reservoir were taken from stations X1 to X5 each month in 2017 (Figure 1). Because the depths at stations X1 and X2 were very shallow (<1.3 m), only surface (0.5 m) water (1000 ml) was collected there. Stations X3 and X5 were located in the deepest parts of the reservoir, with a depth of approximately 30 m, and station X5 was also near the dam where the water intake was positioned. Therefore, water samples (1000 ml) at the surface and 3, 5, 7, 10, 13, 15, 17, 20, 25, and 30 m below the surface were collected. The water at station X4 had a depth of approximately 16 m, and water samples (1000 ml) at 0.5, 5, 10, and 15 m below the surface were collected. The water columns at stations Y2 and Y4 were very shallow (<1.5 m), therefore, only surface water was collected there.

The water temperature, pH and dissolved oxygen (DO) profiles were measured *in situ* using a multi-parameter water quality meter EXO2 (YSI, United States). Water samples for chemical and isotope analysis were collected in pre-sterilized 1 l polyethylene bottles in triplicate from these stations. The samples were transported to the laboratory as soon as possible and stored at 4°C. Before analysis, the water samples were filtered through pre-combusted (450°C, 2 h) GF/F filters.

Water samples for determining the phytoplankton species were collected in triplicate in 1 l polyethylene bottles. The samples were collected from the surface to a depth of 30 m in the reservoir. The samples of phytoplankton were preserved with acidic Lugol’s iodine solution (2% final concentration) *in situ* for later enumeration using a sedimentation technique. Phytoplankton samples were stored in polypropylene vials at 4°C before taxonomical analysis.

Quantitative zooplankton samples were collected as a 5 l water samples at each station from the surface to a depth of 30 m in the reservoir. Samples were filtered through a mesh of 64 μm to form an integrated sample. Vertical hauls were also made with 64 and 113 μm meshes. These zooplankton samples were preserved with 4% formaline and stored in polypropylene vials at 4°C before taxonomical analysis.

Total nitrogen (TN), NO_3_^–^-N, and NH_4_^+^-N analyses were carried out using standard combined persulfate digestion methods for water quality (American Public Health Association [APHA] et al., 1989). The chlorophyll *a* (Chl *a*) concentrations were determined by a spectrofluorometer (Hitachi U-2810, United States) after extraction in 90% ethanol and filtering with glass microfiber filters (Whatman GF/F) (Pápista et al., 2002).

The phytoplankton samples were settled for 48 h by adding Lugol’s solution, and gradually enriched to 10 ml. The cell density was measured with a Sedgwick-Rafter counting chamber under a fluorescence microscope at 200–400× magnification (Olympus, Japan). At least 100 individual cells from every abundant taxon were counted in each sample. Phytoplankton species were identified, as suggested by Hu and Wei (2006).

The identification of zooplankton was carried out by qualified Aquatic Biology Center (Jinan University, Guangzhou). The samples were counted (75× magnification) and taxonomically identified (150× magnification) as Rotifera, Cladocera, and Copepoda species (Wang, 1961; Chiang and Du, 1979; Shen, 1979), using a stereo microscope (Nikon, Japan).

### Algal Strain and Culture Conditions

Strains of *Microcystis densa* (No. MD-1) and *Staurodesmus aristiferus* (No. SA-1) were isolated from the water column of Lianhe Reservoir in July and January 2017, respectively, and were maintained in the algal collection at the School of Environmental Science and Technology, Sun Yat-sen University, China.

Prior to the experiment, the cultures were re-inoculated three times during the exponential phase in BG-11 media (Stanier et al., 1971) and had final nitrogen concentrations of approximately 4 mg l^–1^. The cultures were maintained at 28 ± 1°C for *M. densa* and at 15 ± 1°C for *S. aristiferus*, in a light dark cycle of 12:12 with an irradiation of 100 μmol photons m^–2^ s^–1^. Antibiotics including penicillin G and streptomycin sulfate, were used to exclude bacterial contamination 48 h before the next inoculation (Guillard, 1973). The cultures were checked for bacterial contamination by 4′,6-diamidino-2-phenylindole (DAPI) (Sigma) staining at regular intervals by microscopic inspection.

### Laboratory Experiments

The initial cell densities of *M. densa* and *S. aristiferus* in the media were both ∼4.5 × 10^5^ cells ml^–1^. NO_3_^–^-N was added as the substrate with final concentrations of ∼1.4 mg N l^–1^. To simulate actual field conditions as much as possible, the treatments were incubated at 28 ± 1°C for *M. densa* and at 15 ± 1°C for *S. aristiferus* with an irradiation of 100 μmol photons m^–2^ s^–1^. These conditions were consistent with the actual water temperature and light intensity recorded in the field. These treatments were performed in triplicate.

Samples for the cell counts were obtained daily with fixation in a 2% acid Lugol’s solution. Cell density was measured with a Sedgwick-Rafter counting chamber under a light inverted microscope (Olympus, Japan). The specific growth rates (μ, d^–1^) of *M. densa* and *S. aristiferus* were calculated according to the following equation:

(1) $$\mu =\frac{\ln N_{2}-\ln N_{1}}{t_{2}-t_{1}}$$

where *N*_2_ and *N*_1_ were the cell densities at respective time, *t*_2_ and *t*_1_.

Samples for the Chl *a* and NO_3_^–^-N analyses were obtained daily in triplicate. Fifty ml Chl *a* samples were determined by a spectrofluorometer (Hitachi U-2810, United States) after extraction in 90% ethanol and filtering with glass microfiber filters (Whatman GF/F) (Pápista et al., 2002). Hundred ml NO_3_^–^-N samples were filtered through pre-combusted (450°C, 2 h) GF/F filters and measured according to the APHA method (1989).

### Stable Isotope Ratios for the PN and TDN Analyses

One liter and 250 ml water samples were required for δ^15^N_PN_ measurements of the field and laboratory water samples, respectively. The samples were filtered through precombusted (450°C, 2 h) GF/F filters. After drying at 60°C for 48 h, the cell particulate samples for δ^15^N_PN_ were scraped off the membrane with a small blade. The samples were stored in tin capsules for measurement.

One liter and 250 ml water samples were also filtered through precombusted (450°C, 2 h) GF/F filters for δ^15^N_TDN_ measurements of the field and laboratory samples, respectively. These water samples for δ^15^N_TDN_ were freeze-dried at −80°C for 72 h to a powder and stored in tin capsules for measurement.

Three sediment cores were taken at each station in the reservoir for δ^15^N_PN_ measurements using a Mackereth corer (Mackereth, 1969). These sediments were retrieved from a depth of 0 ∼ 5 cm (Glew et al., 2001). Three replicate sediment cores were composited into one sample. After sampling, the cores were subsampled in the laboratory at 1 cm intervals. These sediment samples were freeze-dried at −80°C for 72 h. The dried sediment samples were then ground in an agate grinder and sieved through a 0.149 mm mesh, and stored in tin capsules until isotope analysis.

All δ^15^N_P__*N*_ and δ^15^N_TDN_ samples were measured by continuous flow isotope ratio mass spectrometry using a Flash 2000 elemental analyzer coupled to a Thermo Fisher Delta Plus XP IRMS (Thermo Fisher, United States). The results were expressed in the delta notation as follows:

(2) δ15N(‰)=[(N15Nsample14N15Nreference14)-1]×103

The δ^15^N_PN_ measurements were calibrated against the international standard of atmospheric N_2_. The standard deviation (S.D.) for the analytical standards was approximately ± 0.2‰.

### Statistical Analysis

A one-way ANOVA with a Tukey test was performed to compare the differences and correlations among each parameter. A *P*-value < 0.05 was regarded as significant and <0.01 as highly significant for all tests. Principal component analysis (PCA) was used to determine the environmental variables that explain the largest per cent variance in δ^15^N_PN_ and δ^15^N_TDN_. Prior to the analysis, untransformed data in all cases were tested for normality and homogeneity of variation. Statistical analyses was performed using the SPSS 19.0 statistical package for personal computers (SPSS, United States).

## Results

### Spatial and Temporal Variations in the Hydrographic and Biogeochemical Properties Within the Reservoir

**FIGURE 2:**
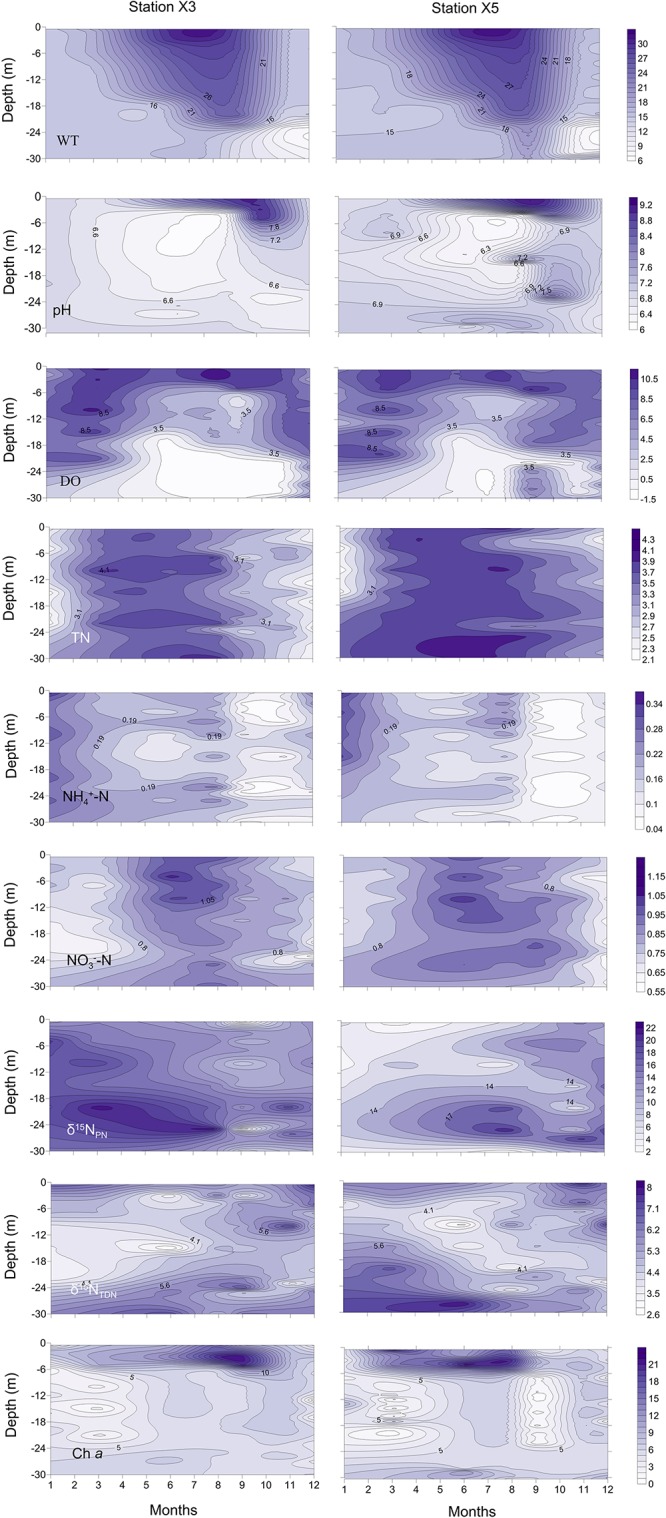
Vertical profiles of the water temperature (WT, °C), pH, dissolved oxygen (DO, mg l^– 1^), total nitrogen (TN, mg l^– 1^), ammonia (NH_4_^+^-N, mg l^– 1^), nitrate (NO_3_^–^-N, mg l^– 1^), particulate nitrogen stable isotope ratios (δ^15^N_PN_, ‰), total dissolved nitrogen stable isotope ratios (δ^15^N_TDN_, ‰) and chlorophyll *a* (Chl *a*, μg l^– 1^) at stations X3 and X5 in 2017 in the Lianhe Reservoir.

Thermal stratification occurred from March to November (the thermal stratification period) and was absent in December, January and February (the mixing period) when the vertical temperature difference in the water column was small (Figure 2, WT). During the thermal stratification period, the water temperature (WT) was high, ranging from 27.3 ± 0.1 to 32.2 ± 0.1°C in the epilimnion but approximately 9.3 ± 0.2 to 12.4 ± 0.1°C in the hypolimnion. The thermocline was at a depth of approximately 20 m. During the mixing period, the WT was approximately 10°C in the whole water column.

The pH values were significantly higher during the thermal stratification period than during the mixing period (*p* < 0.05) (Figure 2, pH). The values from the surface to a depth of 6 m (7.2 ± 0.3 ∼ 9.2 ± 0.2) were significantly higher than those in other layers during the thermal stratification period, indicating a distinct stratified state (*p* < 0.05). The pH values (approximately 6.9 ± 0.3) were not stratified during the mixing period.

In the surface of the water column, the DO content was sufficient, ranging from 5.9 ± 0.4 to 11.2 ± 0.2 mg l^–1^. During the thermal stratification period, the epilimnion was even hypersaturated at times (from 8.5 ± 0.3 to 10.5 ± 0.2 mg l^–1^), but the DO content was below 2.5 ± 0.2 mg l^–1^ in the hypolimnion. Especially from June to November, DO concentrations in the hypolimnion can be as low as 0 mg l^–1^, forming an anoxic closed region. The oxycline occurred within the thermocline at a depth of approximately 16–20 m. DO was relatively abundant at the bottom during the mixing period. Vertical DO stratification was not obvious (Figure 2, DO).

The concentrations of TN were not vertically stratified year round (Figure 2, TN). The concentrations were approximately 3.1 ± 0.4 to 4.3 ± 0.2 mg l^–1^ during the thermal stratification period and 2.1 ± 0.3 to 3.1 ± 0.1 mg l^–1^ during the mixing period. TN concentrations during the thermal stratification period were higher than those during the mixing period (*p* < 0.05).

The concentrations of NO_3_^–^-N were much higher than the concentrations of NH_4_^+^-N, indicating that NO_3_^–^-N is the primary source of inorganic nitrogen in the reservoir (Figure 2, NO_3_^–^-N and NH_4_^+^-N). The NO_3_^–^-N concentration during the thermal stratification period was approximately 0.75 ± 0.01 ∼ 1.20 ± 0.01 mg l^–1^, which was significantly higher than that (0.55 ± 0.02 ∼ 0.75 ± 0.01 mg l^–1^) during the mixing period (Figure 2, NO_3_^–^-N) (*p* < 0.05). The highest concentrations of NH_4_^+^-N (0.34 ± 0.03 mg l^–1^) were detected in January, while the lowest concentrations (0.04 ± 0.01 mg l^–1^) occurred from September to November (Figure 2, NH_4_^+^-N). Neither the NO_3_^–^-N nor the NH_4_^+^-N concentrations changed significantly with depth (*p* > 0.05).

During the thermal stratification period, the vertical profile of δ^15^N_PN_ varied dramatically (Figure 2, δ^15^N_PN_). The δ^15^N_PN_ values in the hypolimnion (16.3 ± 0.3 ∼ 22.7 ± 0.5‰) were significantly higher than those in the epilimnion (2.0 ± 0.1 ∼ 16.4 ± 0.3‰) (*p* < 0.05). During the mixing period, the δ^15^N_PN_ value varied little with depth (Figure 2, δ^15^N_PN_).

The values of δ^15^N_TDN_ were significantly lower than the values of δ^15^N_PN_ (*p* < 0.05), ranging from 2.6 ± 0.3 to 8.0 ± 0.4‰ (Figure 2, δ^15^N_TDN_). The δ^15^N_TDN_ values were approximately 5.2 ± 0.2 ∼ 7.9 ± 0.1‰ at the surface and approximately 4.6 ± 0.2 ∼ 8.0 ± 0.4‰ at the bottom. The average value of δ^15^N_TDN_ during the mixing period (3.7 ± 0.3‰) was higher than that during the thermal stratification period (6.1 ± 0.1‰) (Figure 2, δ^15^N_TDN_).

Chl *a*, a common pigment in phytoplankton cells, is often used as an environmental index to characterize phytoplankton biomass. The Chl *a* concentration was high (6.1 ± 0.2 ∼ 22.3 ± 0.2 μg l^–1^) from the surface water to a depth of 6 m, where most phytoplankton gathered (Figure 2, Chl *a*). The average concentration of Chl *a* during the thermal stratification period (12.3 ± 0.4 μg l^–1^) was higher than that during the mixing period (4.6 ± 0.2 μg l^–1^). The Chl *a* concentration was distributed evenly in the vertical direction during the mixing period (Figure 2, Chl *a*).

### Phytoplankton Community and the Dominant Species

A total of 87 and 80 phytoplankton species were identified during the thermal stratification and mixing periods, respectively (Supplementary Table S1). *M. densa* and *S. aristiferus* were the dominant species during the thermal stratification and mixing periods in 2017, respectively (Table 2). During the mixing period, the maximum cell density of *S. aristiferus* was 6.3 ± 0.2 × 10^5^ cells ml^–1^ in the surface water (January 2017). However, during the thermal stratification period, the maximum cell density of *M. densa* was 9.6 ± 0.2 × 10^5^ cells ml^–1^ and occurred in the epilimnion, while *S. aristiferus* was still the dominant species in the hypolimnion, with a maximum cell density of 1.4 ± 0.1 × 10^5^ cells ml^–1^ (August, 2017). The total cell densities of phytoplankton during the thermal stratification period were much higher than those during the mixing period (Table 2). The cell densities of dominant species accounted for 77 to 93% of the total cell densities of phytoplankton (Table 2).

**TABLE 2:** Dominant species of phytoplankton and their cell densities/total cell densities of phytoplankton (×10^5^ cells ml^–1^) at stations X3 and X5 at a water layer depth of 0.5, 10, and 20 m during thermal stratification (e.g., August) and mixing periods (e.g., January) in 2017 at Lianhe Reservoir.

**Depth (m)**	**Mixing period (e.g., January)**		**Thermal stratification period (e.g., August)**	
		
	**X3**	**X5**	**X3**	**X5**
0.5	*S. aristiferus*	*S. aristiferus*	*M. densa*	*M. densa*
	(6.3 ± 0.2/7.3 ± 0.3)	(5.2 ± 0.3/5.7 ± 0.4)	(9.6 ± 0.2/10.3 ± 0.3)	(8.9 ± 0.3/9.7 ± 0.4)
10	*S. aristiferus*	*S. aristiferus*	*M. densa*	*M. densa*
	(3.1 ± 0.2/3.8 ± 0.2)	(1.7 ± 0.1/2.2 ± 0.2)	(3.2 ± 0.1/3.7 ± 0.3)	(2.6 ± 0.1/2.8 ± 0.1)
25	*S. aristiferus*	*S. aristiferus*	*S. aristiferus*	*S. aristiferus*
	(1.3 ± 0.1/1.4 ± 0.1)	(1.0 ± 0.0/1.3 ± 0.1)	(1.3 ± 0.1/1.5 ± 0.1)	(1.4 ± 0.1/1.7 ± 0.0)

*The values are the mean ± SD (*n* = 3).*

### Zooplankton Community

A total of 4 rotifera, 3 cladocera and 2 copepoda species were found during the thermal stratification period (Supplementary Table S2). During the mixing period, there were 7 Rotifera, 3 Cladocera and 3 Copepoda species in the reservoir (Supplementary Table S2). Zooplankton densities varied from 2 to 19 ind. l^–1^ and 7 to 27 ind. l^–1^ during thermal stratification and mixing periods, respectively, indicating that zooplankton were more abundant during the mixing period.

### δ^15^N_PN_ Values in Rivers and Sediments

The δ^15^N_PN_ values from the surface water at stations X1 and X2 and the stations in rivers (Y2 and Y4) are also shown in Table 3. The δ^15^N_PN_ values at X1 and X2, the two stations in the reservoir that were the closest to the river shore, varied from 4.9 ± 0.4 to 7.4 ± 0.2‰ and from 4.5 ± 0.3 to 6.9 ± 0.7‰, respectively. The δ^15^N_PN_ values at Y2 and Y4, the two stations in the rivers and close to the reservoir, ranged from 1.4 ± 0.3 to 5.7 ± 0.3‰ and from 4.2 ± 0.4 to 5.7 ± 0.4‰, respectively. The δ^15^N_PN_ values from the rivers (Y2 and Y4) were much lower than those at reservoir stations X1 and X2 (Table 3).

**TABLE 3:** Seasonal variations of particulate nitrogen stable isotope ratios (δ^15^N_PN_, ‰) in surface water of stations X1 and X2 in the reservoir, and stations Y2 and Y4 in the nearby rivers in 2017.

**Months**	**X1**	**X2**	**Y2**	**Y4**
January	6.9 ± 0.3	5.6 ± 0.6	5.2 ± 0.3	5.4 ± 0.5
February	7.0 ± 0.3	5.4 ± 0.1	4.8 ± 0.4	4.8 ± 0.9
March	7.4 ± 0.2	6.1 ± 0.3	5.5 ± 0.5	5.7 ± 0.4
April	6.2 ± 0.4	5.1 ± 0.2	4.4 ± 0.1	4.2 ± 0.3
May	5.9 ± 0.3	4.7 ± 0.4	3.2 ± 0.2	4.7 ± 0.4
June	5.7 ± 0.6	4.5 ± 0.3	1.4 ± 0.3	5.4 ± 0.6
July	5.7 ± 0.3	5.2 ± 0.4	2.3 ± 0.2	4.3 ± 0.2
August	6.0 ± 0.4	5.7 ± 0.3	4.1 ± 0.4	4.4 ± 0.2
September	4.9 ± 0.4	6.9 ± 0.7	5.7 ± 0.3	4.2 ± 0.4
October	5.0 ± 0.4	6.5 ± 0.8	2.8 ± 0.3	4.8 ± 0.7
November	6.1 ± 0.2	5.3 ± 0.2	4.4 ± 0.3	5.5 ± 0.2
December	6.7 ± 0.4	5.7 ± 0.3	2.5 ± 0.4	4.7 ± 0.6

*The values are the mean ± SD (*n* = 3).*

The δ^15^N_PN_ values of sediments at different depths at stations X1 ∼ X5 were also measured (Table 4). There were small differences in these δ^15^N_PN_ values among depths and stations (*p* > 0.05). The average value of δ^15^N_PN_ was 3.0 ± 0.5 ‰. However, the δ^15^N_PN_ values in the sediment (2.3 ± 0.2 ∼ 3.8 ± 0.2‰) were much lower than those in the bottom of the water column, especially at station X3 (*p* < 0.05).

**TABLE 4:** Nitrogen stable isotope ratios (δ^15^N_PN_, ‰) at different depths of sediments at stations X1, X2, X3, X4, and X5 in Lianhe Reservoir.

**Depths (cm)**	**X1**	**X2**	**X3**	**X4**	**X5**
1	3.4 ± 0.3	2.7 ± 0.2	2.4 ± 0.2	2.7 ± 0.4	2.5 ± 0.3
2	3.0 ± 0.5	2.9 ± 0.3	3.2 ± 0.4	2.4 ± 0.2	2.3 ± 0.2
3	2.8 ± 0.4	3.1 ± 0.3	3.3 ± 0.1	3.8 ± 0.2	2.6 ± 0.1
4	3.2 ± 0.1	3.3 ± 0.3	3.6 ± 0.2	3.5 ± 0.2	2.7 ± 0.2
5	2.4 ± 0.1	2.8 ± 0.1	2.5 ± 0.1	3.4 ± 0.1	3.3 ± 0.2

*The values are the mean ± SD (*n* = 3).*

### The Relationships Between δ^15^N_PN_, δ^15^N_TDN_ and Phytoplankton in the Field

**FIGURE 3:**
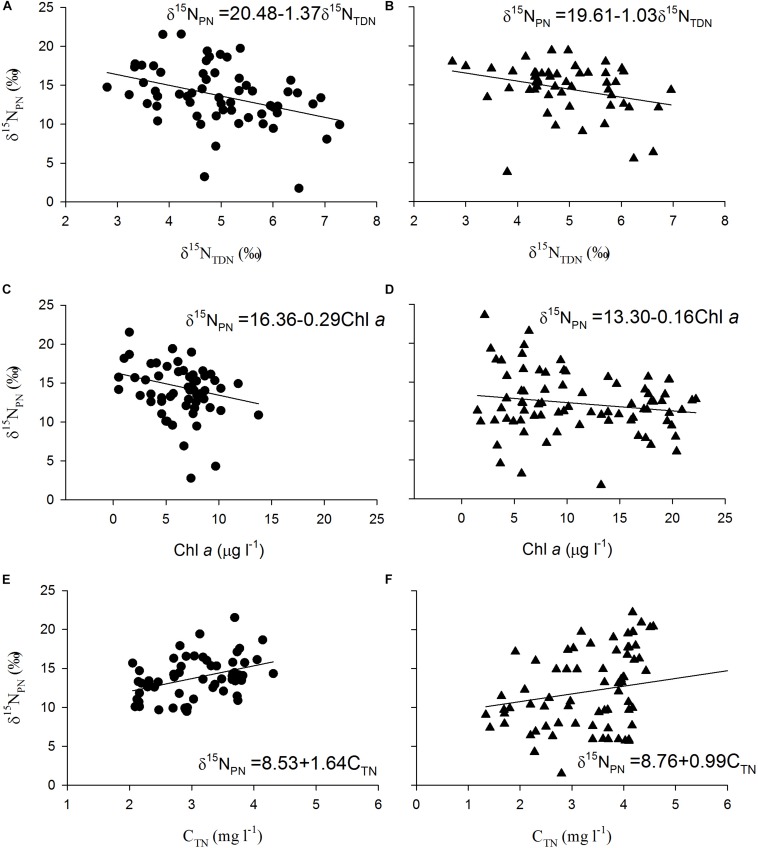
Scatter plots of particulate nitrogen stable isotope ratios (δ^15^N_PN_, ‰) against total dissolved nitrogen stable isotope ratios (δ^15^N_TDN_, ‰) during **(A)** mixing and **(B)** thermal stratification periods, Chlorophyll *a* (Chl *a*) during **(C)** mixing and **(D)** thermal stratification periods, and total nitrogen (C_*TN*_) during **(E)** mixing and **(F)** thermal stratification periods as well as their fitted curves in every depth at stations X1 ∼ X5 in Lianhe Reservoir in 2017.

The δ^15^N_PN_ values in the reservoir were negatively correlated with the δ^15^N_TDN_ and Chl *a* concentrations (Figures 3A–D) and positively correlated with the TN concentrations (Figures 3E,F) in both the thermal stratification and mixing periods. These results indicate that phytoplankton biomass is potentially the primary factor affecting the variations in δ^15^N_PN_ in the reservoir.

**FIGURE 4:**
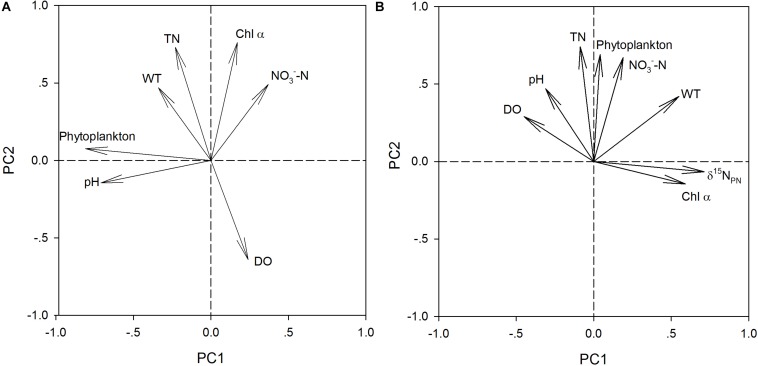
Principal component 1 (PC1) versus principal component 2 (PC2) for δ^15^N_PN_ **(A)** and δ^15^N_TDN_ **(B)** using monthly values at eat depth of stations X1 ∼ X5 in the Lianhe Reservoir in 2017.

PCA of δ^15^N_PN_, δ^15^N_TDN_ and the associated environmental variables revealed three principal components (PCs) with eigenvalues greater than 1.0 (Table 5). The first principal component (PC1) for δ^15^N_PN_ included phytoplankton cell densities and pH, and explained 45.2% of the variance in the dataset. The second principal component (PC2) for δ^15^N_PN_ included DO, TN and Chl *a*, and explained 26.3% of the variance. The third principal component (PC3) included NO_3_^–^-N and WT, and explained 18.1% of the variance (Table 5). These three PCs together accounted for 89.6% of the variation for δ^15^N_PN_ (Figure 4A). For δ^15^N_TDN_, PC1 contained δ^15^N_PN_ and Chl *a*, and explained 34.1% of the variance. PC2 contained phytoplankton cell density, NO_3_^–^-N and TN, and explained 26.2% of the variance. PC3 contained WT, DO, and NH_4_^+^-N and explained 15.9% of the variance (Table 5). These three PCs together accounted for 76.2% of the variance in δ^15^N_TDN_ (Figure 4B). However, δ^15^N_PN_ and δ^15^N_TDN_ were interacted with each other. δ^15^N_PN_ can not be considered as a factor influencing δ^15^N_TDN_. Moreover, both Chl *a* (represents phytoplankton biomass) and phytoplankton cell density indicated the phytoplankton abundance. Therefore, these two factors were equally important for variations in δ^15^N_TDN_. These results showed that phytoplankton cell density was the primary factor controlling the variations in δ^15^N_PN_ and δ^15^N_TDN_.

**TABLE 5:** Principal components and eigenvalues for δ^15^N_PN_ and δ^15^N_TDN_ in Lianhe Reservoir based on the analysis using monthly values at each depth of stations X1 ∼ X5 in 2017.

	**Component**	**Variables**	**Eigenvalues**		
			
			**Total**	**% of Variance**	**Cumulative%**
δ^15^N_PN_	1	Phytoplankton cell density, pH	2.9	45.2	45.2
	2	Chl *a*, TN, DO	1.7	26.3	75.1
	3	NO_3_^–^, WT	1.3	18.1	89.6
δ^15^N_TDN_	1	δ^15^N_PN_, Chl *a*	2.7	34.1	34.1
	2	NO_3_^–^, TN, phytoplankton cell density	2.1	26.2	60.3
	3	WT, DO, NH_4_^+^	1.3	15.9	76.2

### Variations in δ^15^N_PN_ and δ^15^N_TDN_ in Cultures of *M. densa* and *S. aristiferus*

**FIGURE 5:**
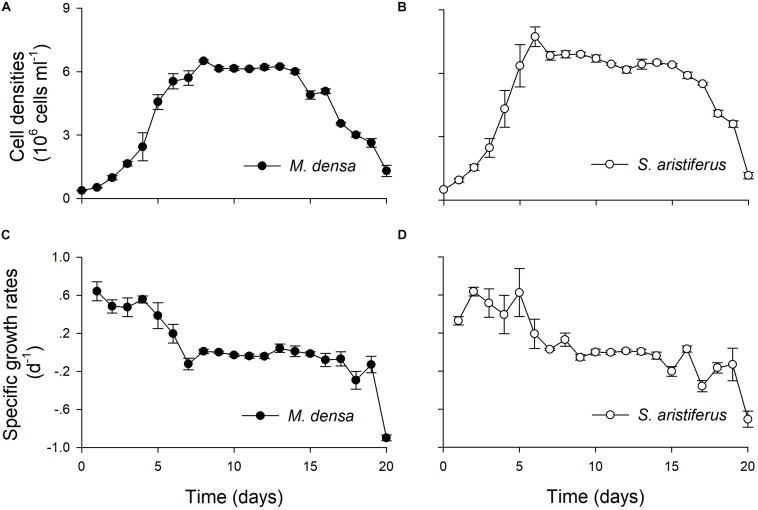
The growth of *M. densa* and *S. aristiferus* during treatment with NO_3_^–^-N. **(A)** Cell densities of *M. densa*. **(B)** Cell densities of *S. aristiferus*. **(C)** Specific growth rates of *M. densa*. **(D)** Specific growth rates of *S. aristiferus*. The values are the mean ± SD (*n* = 3).

Both *M. densa* and *S. aristiferus* grew well, and the cell densities peaked on Days 5 ∼ 6 in the experiments (Figures 5A,B). The maximum cell density of *M. densa* was 6.9 ± 0.5 × 10^6^ cells l^–1^, while that of *S. aristiferus* was 7.8 ± 0.4 × 10^6^ cells l^–1^. Both *M. densa* and *S. aristiferus* cells entered the stationary phase after Day 7 and decreased in number after Day 17. The specific growth rates of *M. densa* decreased on Day 4, while that of *S. aristiferus* decreased on Day 5 (Figures 5C,D).

**FIGURE 6:**
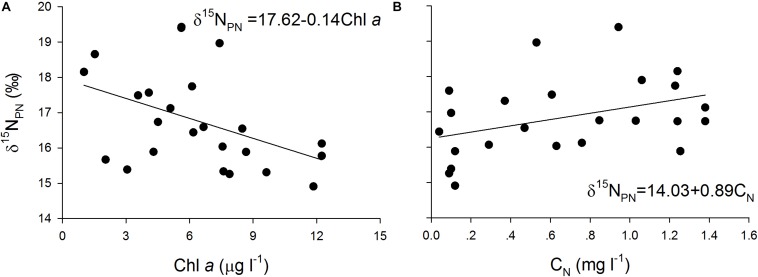
Scatter plots of particulate nitrogen stable isotope ratios (δ^15^N_PN_, ‰) against **(A)** Chlorophyll *a* (Chl *a*, μg l^– 1^) and **(B)** NO_3_^–^-N (C_*N*_, mg l^– 1^) and their fitted curves in the batch cultures.

The δ^15^N_PN_ values were negatively correlated with the Chl *a* concentration (Figure 6A) and positively correlated with the nitrogen concentrations (Figure 6B) in the cultures. Similar trends were found in the field (Figure 3), indicating that variations in δ^15^N_PN_ in the reservoir were mainly determined by phytoplankton cell density, especially the dominant species cell density.

**FIGURE 7:**
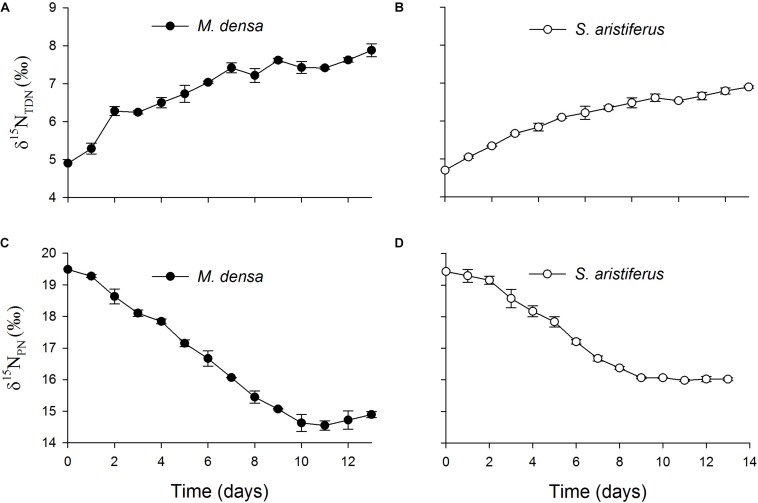
The variation of the total dissolved nitrogen stable isotope ratios (δ^15^N_TDN_) in the batch cultures of **(A)** *M. densa* and **(B)** *S. aristiferus*, and the particulate nitrogen stable isotope ratios (δ^15^N_PN_) in the batch cultures of **(C)** *M. densa* and **(D)** *S. aristiferus*. The values are the mean ± SD (*n* = 3).

The values of δ^15^N_TDN_ in the cultures of both *M. densa* and *S. aristiferus* increased (Figures 7A,B). In the stationary phase on Day 13, the maximum value of δ^15^N_TDN_ was 7.9 ± 0.3‰ in the culture of *M. densa*, while that in the culture of *S. aristiferus* was 6.9 ± 0.2‰. However, the δ^15^N_PN_ value decreased continuously and reached its lowest value on Day 11: 14.6 ± 0.3‰ in *M. densa* cultures and 16.0 ± 0.1‰ in *S. aristiferus* cultures (Figures 7C,D) indicating that the continuous growth of both *S. aristiferus* and *M. densa* can directly reduce δ^15^N_PN_ and increase δ^15^N_TDN_.

## Discussion

### Factors Affecting Variations in δ^15^N_PN_ and δ^15^N_TDN_

In general, nitrogen in reservoirs can be divided into exogenous nitrogen, including that entering via atmospheric deposition and rivers, and endogenous nitrogen, which originates from denitrification, sediments and biological processes. These nitrogen sources influence variations in δ^15^N_PN_ and δ^15^N_TDN_ in reservoirs to different degrees.

#### Exogenous Factors

Nitrogen pollutants in the air, mainly nitrate and nitrite, enter the surface water of the reservoir by wet deposition. Because the nitrogen isotope fractionation in the atmosphere is different from that in the water column, atmospheric deposition changes the variation in δ^15^N in the water column. However, the δ^15^N_TDN_ values in the surface water of Lianhe Reservoir ranged from 4.5 ± 0.3 ∼ 7.4 ± 0.2‰, which are much different from of the values for wet deposition (Xiao and Liu, 2002; Elliott et al., 2007). This result indicated that nitrogen from atmospheric deposition made little contribution to the nitrogen stable isotope composition in Lianhe Reservoir.

Particulate matter from the shore can be washed into the reservoir via rivers during heavy rain events. However, our results showed that the δ^15^N_PN_ in the rivers near the reservoir (stations Y2 and Y4) was lower than that in the surface water at the junctions of Lianhe Reservoir and the nearby rivers (stations X1 and X2) (Table 3). If nitrogen from rivers is the major controller of variation in δ^15^N_PN_ in Lianhe Reservoir, the river δ^15^N_PN_ value should be greater than or equal to the values in the reservoir. However, our investigation showed the opposite, suggesting that the effects of nitrogen from rivers on the variations in the δ^15^N_PN_ values in the reservoir were not large as expected.

#### Endogenous Factors

Isotopic fractionation occurs during nitrogen migration and transformation. Sugimoto et al. (2010, 2014) suggested that the value of δ^15^N_PN_ is principally determined by the dissolved inorganic nitrogen (DIN) concentration. Hou et al. (2013) suggested that TN is the primary driver of the change in δ^15^N_PN_. However, in our study, the vertical profiles of TN, NO_3_^–^-N, and NH_4_^+^-N changed little, but particulate and dissolved nitrogen stable isotope fractionation had already occurred (Figure 2). PCA revealed that the effects of TN, NO_3_^–^-N, and NH_4_^+^-N on both δ^15^N_PN_ and δ^15^N_TDN_ were not significant (Table 5). Obviously, nitrogen concentration might affect the variations in δ^15^N_PN_ and δ^15^N_TDN_, but it was not the critical controlling factor.

Debris from shore or sediment is generally considered an important source of δ^15^N_PN_, especially in deep water (Hamilton et al., 2001; Wollheim et al., 2001). However, in Lianhe Reservoir, the δ^15^N_PN_ values in the sediments were all lower than those in the water column (Tables 3, 4). Furthermore, we observed a large proportion of algal cells in particulate matter (PM) under a microscope. Therefore, we believe that Lianhe Reservoir is a phytoplankton-dominated PM subtropical reservoir. Other surveys have also shown that lakes or reservoirs with strong solar radiation and thermal stratification of the water body are usually phytoplankton-dominated PM pools (Gu, 2009). Lianhe Reservoir, located in southern China, is exposed to intense solar radiation. The water column in the reservoir thermally stratifies in summer. Therefore, the main source of PM is not the debris but phytoplankton cells.

Sediments contributed a low δ^15^N_PN_ level (2.3 ± 0.2 ∼ 3.6 ± 0.2‰) in Lianhe Reservoir, which was significantly lower than the δ^15^N_PN_ in the bottom water (*p* < 0.05) (Figure 2 and Table 4). This difference suggested that the denitrification between sediment and the water column at the bottom was not strong. Other studies have shown that the δ^15^N_PN_ values resulting from denitrificatin in sediments are approximately 14 ∼ 38‰ (Casciotti et al., 2003), which were very different from the values obtained in this study. We speculate that the reason for this result is that reservoirs are semiartificial water bodies. The reservoir management station dredges every year, and the community of denitrobacteria in the sediment is destroyed. Furthermore, sedimentary denitrification had a minimal effect on the vertical variations in δ^15^N_PN_ during the thermal stratification period because transport limited the overall rate, resulting in a low isotopic fractionation potential (Hadas et al., 2009). However, we cannot rule out the possibility that the remineralization of organic nitrogen and subsequent nitrification might have contributed to δ^15^N_TDN_ in the reservoir during the mixing period, which requires further study.

Biochemical processes must be considered when studying the variations in nitrogen stable isotopes in reservoirs. In our study, although the distribution of δ^15^N_PN_ differed greatly between the thermal stratification and mixing periods, δ^15^N_PN_ still showed a strong correlation with phytoplankton cell density in both the field investigation and laboratory experiments (Figures 4, 6). PCA showed that variations in δ^15^N_PN_ and δ^15^N_TDN_ were principally determined by phytoplankton cell density (Table 5). These results suggested that phytoplankton cell density was the key factor controlling nitrogen stable isotope composition. Phytoplankton biological effects are a primary factor in the N cycle of subtropical reservoirs, and their effects are much greater than those of other physical and chemical factors.

The laboratory experimental results showed that the δ^15^N_PN_ values decreased and the δ^15^N_TDN_ values increased in both *M. densa* and *S. aristiferus* cultures (Figure 7). To simulate the natural dynamics, phytoplankton were cultured at different WT, and the results were consistent with those of our field investigation. Because of the different culture temperatures, we could not compare the nitrogen fractionation effects between these two dominant species, but the results still suggested that both of these dominant species could regulate the assignment of ^15^N between particular nitrogen and total dissolved nitrogen. Based on the fact that both of these dominant species had the same regulatory capacity, this ^15^N assignment mechanism seemed to be closely related to only cell density, not phytoplankton species. Furthermore, dominant species accounted for 77–93% of the total phytoplankton (Table 2). Therefore, dominant species are an important indicator of total phytoplankton that can be used to evaluate the effect of biological processes on the N cycle.

Zooplankton play a mediating role in the food web of aquatic ecosystems, and excess zooplankton puts predation pressure on phytoplankton (Boyce et al., 2010; De Stasio et al., 2018; Sitta et al., 2018). When zooplankton biomass is low, phytoplankton will proliferate rapidly (Paerl et al., 2011; Er et al., 2018). In our study, 9 and 13 species of zooplankton were found during the thermal stratification and mixing periods, respectively (Supplementary Table S2). The number of species and the biomass of zooplankton in Lianhe Reservoir were significantly lower than those in other subtropical reservoirs (Lin et al., 2003). On the other hand, phytoplankton cell density could reach up to 10.3 ± 0.3 × 10^5^ cells ml^–1^ (Table 2) and the predation pressure from zooplankton seemed to not have an effect. Therefore, in the plankton community, phytoplankton accounted for an overwhelming proportion of both species and biomass. Zooplankton could surely also regulate the spatial and temporal distribution of δ^15^N. However, the proportion of zooplankton in Lianhe Reservoir was so small that it did not play an important role in controlling δ^15^N_PN_ and δ^15^N_TDN_.

**FIGURE 8:**
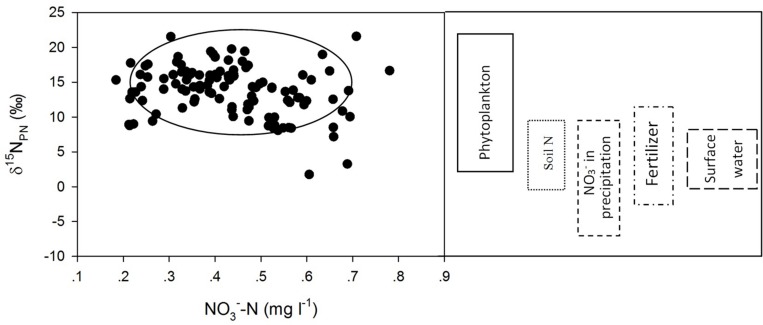
Scatter plots of δ^15^N_PN_ (‰) against NO_3_^–^-N (mg l^– 1^) and the range of δ^15^N_PN_ values from different sources.

Compared to the δ^15^N_PN_ values determined in other studies (Fogg et al., 1998; Kendall, 1998; Russell et al., 1998; Mayer et al., 2002; Vuorio et al., 2006; Bateman and Kelly, 2007; Finlay et al., 2007; Hales et al., 2007; Singleton et al., 2007; Lee et al., 2008; Xue et al., 2009; Doi et al., 2010; Titlyanov et al., 2011; Ólafsson et al., 2013), the δ^15^N_PN_ values in Lianhe Reservoir were within the range of δ^15^N_PN_ values from phytoplankton, which was approximately 3 ∼ 22‰ (Figure 8). This finding also supported our hypothesis that phytoplankton cell density was the primary factor controlling the temporal and spatial distributions of δ^15^N_PN_ in a subtropical reservoir.

### Implications for Biochemical Processes

**FIGURE 9:**
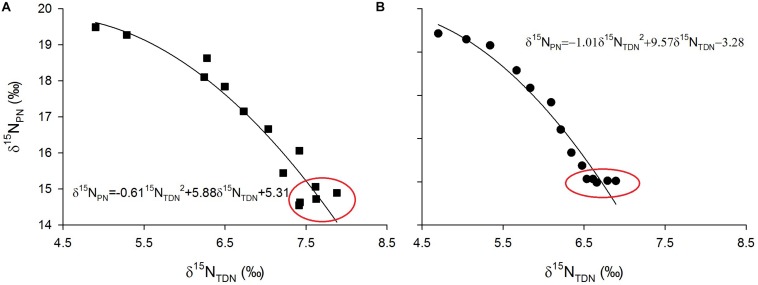
Scatter plots of particulate nitrogen stable isotope ratios (δ^15^N_PN_, ‰) against total dissolved nitrogen stable isotope ratios (δ^15^N_PN_, ‰) and their fitted curves for *M. densa* **(A)** and *S. aristiferus* **(B)** cultures.

Laboratory experiments showed that δ^15^N_PN_ values were inversely proportional to δ^15^N_TDN_ values (Figure 9). Interestingly, in the growth stationary phase, δ^15^N_TDN_ in the media still increased while δ^15^N_PN_ remained relatively constant (red circles in Figure 9). This result indicated that the amount of nitrogen is sufficient for algal cells, which no longer take up nitrogen from the ambient environment. Conversely, algal cells released nitrogen to maintain a balance of intracellular and extracellular nitrogen.

Excessive nitrogen flows into the reservoir and sinks to the bottom, which increases the nitrogen budget year by year. Denitrification is generally considered the major process that removes this excess nitrogen, especially in deep lakes and reservoirs (Han et al., 2014; Zhou et al., 2018). At the anoxic bottom during the thermal stratification period, NO_3_^–^-N reduction to NO_2_^–^-N, N_2_O, and even N_2_ is accomplished by the denitrifying bacterial community (Saunders and Kalff, 2001; Zhang et al., 2019). Generally, the ambient environment at the dark and anoxic bottom is not suitable for phytoplankton. Surprisingly, we found biomass of phytoplankton at the bottom (Table 2). As mentioned above, these phytoplankton not only take up nitrogen but also continuously excrete excess nitrogen into the water column to maintain metabolism stability (Figure 9). That is, in addition to denitrification causing nitrogen loss, there is a process for nitrogen accumulation by phytoplankton. There was no significant difference in the NO_3_^–^-N vertical concentration even during the thermal stratification period (Figure 2), which also supported our deduction. These two opposing pathways maintain the nitrogen balance at the bottom of the reservoir.

Areas with industrial production, urban wastes, and fishing are enriched in δ^15^N_TDN_ and can carry δ^15^N_TDN_ signatures as high as 20‰ (Bedard-Haughn et al., 2003; Leavitt et al., 2006; Hou et al., 2013). However, in the middle water layer (18 ∼ 24 m below the surface) of Lianhe Reservoir, the phytoplankton biomass was low. Additionally, it is difficult for river runoff, rainfall and bottom denitrification to influence the physicochemical processes of this water layer during the thermal stratification period. Moreover, there is also less human activity around the reservoir. Therefore, we speculated that the low δ^15^N_TDN_ value in this layer is close to the background value of δ^15^N_TDN_ in natural subtropical reservoirs. Surely, the value of δ^15^N_TDN_ is also associated with dissolved inorganic and organic nitrogen. Expanding the scope of investigation in further studies is needed to characterize the background δ^15^N_TDN_ signature in natural subtropical reservoirs. However, we believe that the δ^15^N_TDN_ value of this water layer can provide a reference for determining the natural background value of δ^15^N_TDN_.

### Biochemical Characteristics of Subtropical Reservoirs

#### The Thermal Stratification Period

**FIGURE 10:**
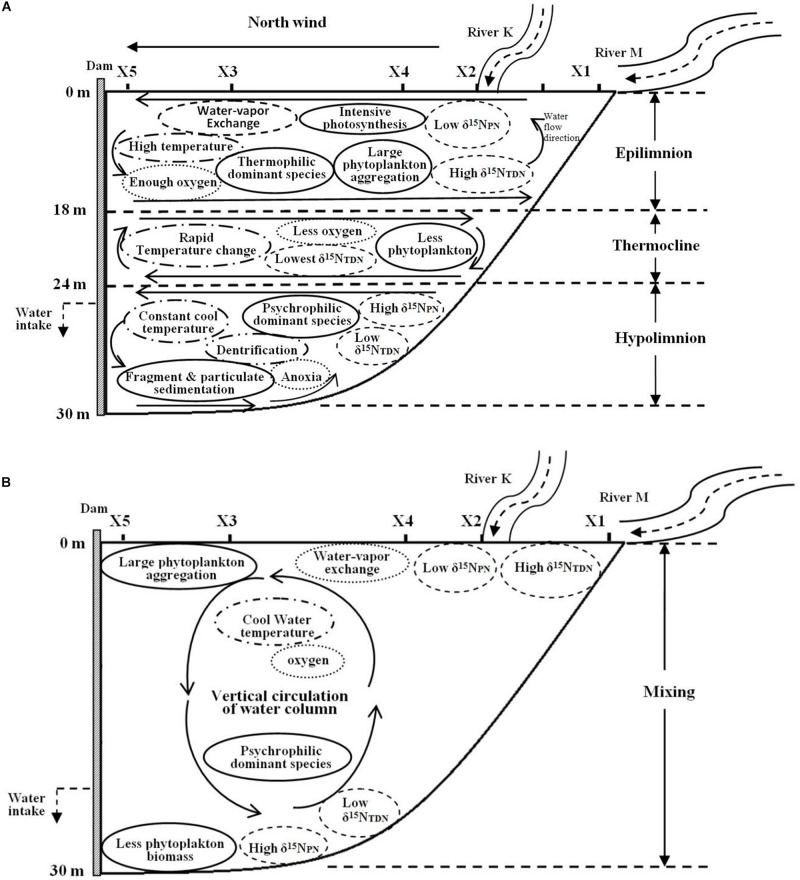
Biochemical characteristics of Lianhe Reservoir during the thermal stratification **(A)** and mixing **(B)** periods.

The depth of the reservoir and intense solar radiation cause a discrepancy in the vertical heat budget during the thermal stratification period. This discrepancy represents a thermocline separating WT in the epilimnion and hypolimnion, leading to the vertical heterogeneity in the biomass and species composition of phytoplankton. The WT in the epilimnion is higher than that in the other layers, especially the surface temperature, which is close to the air temperature. However, the temperature drops sharply in the thermocline, while the temperature in the hypolimnion stays cool year round (Figure 10A).

Because of strong water-vapor exchange at the surface and photosynthesis by phytoplankton, the DO level in the epilimnion is always sufficient and sometimes even supersaturated. However, an anoxic environment is formed because of the lack of oxygen supplementation and phytoplankton respiration in the hypolimnion. There is also an oxycline that usually accompanies the thermocline (Figure 10A).

Due to relatively high temperature and optimum light intensity in the epilimnion, the abundant phytoplankton aggregate for photosynthesis. The dominant species in this layer are thermophilic species, such as *M. densa*. In the thermocline, temperature and light intensity decrease rapidly, and many species cannot endure the severe change in this layer. Therefore, the phytoplankton biomass in the thermocline is less than that in the epilimnion. Even fewer species gather in the hypolimnion, a totally dark anoxic zone. The phytoplankton community structure is mainly composed of psychrophilic species, such as *S. aristiferus*. The cells of dead phytoplankton in the upper layer will be decomposed into many fragments or mineralized into particulates down in the hypolimnion. These fragments and particulates further release nitrogen. Phytoplankton living in the hypolimnion also contributes some nitrogen through metabolism. However, the thermocline prevents the penetration of this nitrogen into the epilimnion, resulting in nitrogen accumulation in the hypolimnion (Figure 10A).

Nitrogen concentrations, such as the TN, NO_3_^–^-N, and NH_4_^+^-N, were higher during the thermal stratification period than during the mixing period. These types of nitrogen have less vertical variation. However, δ^15^N_PN_ and δ^15^N_TDN_ are vertically stratified. Depending on high phytoplankton cell density, the δ^15^N_PN_ value decreases in the epilimnion, while the δ^15^N_TDN_ value increases. In contrast, there are higher δ^15^N_PN_ and lower δ^15^N_TDN_ in the hypolimnion than in the epilimnion due to the lower phytoplankton cell density in the former. Dentrification also occurs in the hypolimnion, removing excessive NO_3_^–^-N and balancing the nitrogen budget (Figure 10A).

#### The Mixing Period

The thermocline disappears during the mixing period. Water density is homogeneous in the vertical direction, resulting in well-mixed water and vertical movement of materials (Figure 10B). Water-vapor exchange still occurs, and there is also sufficient oxygen in the surface water. DO can be transported vertically, forming a homogenous distribution throughout the water column (Figure 10B).

WT cool in the surface water, but solar radiation is still intense. Therefore, many phytoplankton gather in the upper water, and the dominant species changes to a psychrophilic species (*S. aristiferus*). Fewer phytoplankton live at the bottom than in the upper water, which is the same pattern as in the thermal stratification period. The dominant species remain those that prefer to live in cool water. Due to the strong vertical water mixing, particulates and fragments generated from dead algal cells are distributed evenly along the vertical profile.

The concentrations of TN, NO_3_^–^-N, and NH_4_^+^-N are lower in the mixing period than in the thermal stratification period and are still distributed uniformly throughout the water column. Vertical variations in δ^15^N_PN_ and δ^15^N_TDN_ are the same in the mixing and the thermal stratification periods, with lower δ^15^N_PN_ and higher δ^15^N_TDN_ in the upper waters and higher δ^15^N_PN_ and lower δ^15^N_TDN_ at the bottom (Figure 10B). Obviously, variations in δ^15^N_PN_ and δ^15^N_TDN_ are less affected by water movement and WT than by phytoplankton cell density, which dominates.

## Data Availability Statement

The raw data supporting the conclusions of this manuscript will be made available by the authors, without undue reservation, to any qualified researcher.

## Author Contributions

YaC designed the study, carried out the field and indoor experiments, analyzed the results, and drafted the manuscript. CT reviewed and edited the original draft of the manuscript. YiC participated in the field sampling and data interpretation. All authors read and approved the final manuscript.

## Conflict of Interest

The authors declare that the research was conducted in the absence of any commercial or financial relationships that could be construed as a potential conflict of interest.
