# Supplementary material for: Evidence for the Primary Role of Phytoplankton on Nitrogen Cycle in a Subtropical Reservoir: Reflected by the Stable Isotope Ratios of Particulate Nitrogen and Total Dissolved Nitrogen
Source: Front Microbiol. 2019 Sep 25;10:2202. doi: 10.3389/fmicb.2019.02202 (PMC6773875; doi:10.3389/fmicb.2019.02202)
Supplement: Supplementary file 1 [file Table_1.docx]

Table S1 All the phytoplankton species in the Lianhe Reservoir during the thermal stratification and mixing periods in 2017.

| The thermal stratification period | The mixing period |
| --- | --- |
| *Actinastrum fluviatile* | *Anabaena spiroides* |
| *Anabaena spiroides* | *Ankistrodesmus acicularis* |
| *Ankistrodesmus falcatus* | *Ankistrodesmus falcatus* |
| *Ankistrodesmus nivalis* | *Ceratium hirundinella* |
| *Ankistrodesmus spiralis* | *Chroococcus limneticus* |
| *Asterionella japonica* | *Chroomonas acuta* |
| *Ceratium furca* | *Closterium gracile* |
| *Ceratium fusus* | *Closterium moniliforum* |
| *Chroomonas acuta* | *Cocconeis* sp. |
| *Closterium gracile* | *Coelastrum cambricum* |
| *Coelastrum microporum* | *Coelastrum reticulatum* |
| *Coelastrum reticulatum* | *Cosmarium pachydermum* |
| *Coelosphaerium kutzingianum* | *Cosmarium bireme* |
| *Cosmarium bioculatum* | *Crucigenia apiculata* |
| *Cosmarium* sp. | *Crucigenia lauterbornii* |
| *Crucigenia apiculata* | *Crucigenia quadrata* |
| *Crucigenia tetrapedia* | *Crucigenia tetrapedia* |
| *Crucigenia quadrata* | *Cryptomonas ovata* |
| *Cryptomons ovata* | *Cyanobium distomicola* |
| *Cyanobium paarvum* | *Cyclotella meneghiniana* |
| *Cyclotella* sp. | *Cymbella cuspidate* |
| *Cymbella* sp. | *Cymbella gracillis* |
| *Dictyosphaeria cavernosa* | *Cymbella pusilla* |
| *Dictyosphaerium ehrenbergianum* | *Dictyosphaerium ehrenbergianum* |
| *Eudorina elegans* | *Dinobryon bavaricum* |
| *Euglena acus* | *Dinobryon borgei* |
| *Euglena gasterosteus* | *Dinobryon divergens* |
| *Euglena mutabilis* | *Elakatothrix gelatinosa* |
| *Euglena pisciformis* | *Euglena acus* |
| *Euglena polymoeph* | *Euglena mutabilis* |
| *Glenodinium gymnodinium* | *Euglena pisciformi*s |
| *Golenkinia paucispina* | *Euglena polymorpha* |
| *Gomphonema acuminatun* | *Euglena sporogyra* |
| *Gomphonema* sp. | *Fragilaria capucina* |
| *Gonium* sp. | *Glenodinium gymnodinium* |
| *Lepocinclis autumnalis* | *Gomphonema parvulum* |
| *Lepocinclis fusiformis* | *Gomphonema* sp. |
| *Melosira granulata* | *Gonium formosum* Pascher |
| *Melosira* sp. | *Gyrosigma acuminatum* |
| *Melosira varians* | *Gyrosigma* sp. |
| *Merismopedia* sp. | *Hyallotheca dissiiens* |
| *Microcystis densa* | *Kirchneriella contorta* |
| *Microcystis wesenbergii* | *Kirchneriella lunaris* |
| *Navicula bicapitellata* | *Melosira granulata* |
| *Navicula* sp. | *Melosira granulata var. angustissima* |
| *Nitzschia* sp. | *Melosira islandica* |
| *Oscillatoria acuminata* | *Melosira italica* |
| *Oscillatoria chlorine* | *Melosira varians* |
| *Pandorina morum* | *Melosira wrians* |
| *Pediastrum clathratum* | *Micractinium pusillum* |
| *Pediastrum duplex* | *Microcystis densa* |
| *Pediastrum duplex var.clathratum* | *Microcystis wesenbergii* |
| *Pediastrum duplex var.gracillimum* | *Navicula bicapitellata* |
| *Peridinium gatunense* | *Navicula cuspidata* |
| *Peridinium* sp. | *Navicula.*sp. |
| *Phacus acuminatus* | *Oocystis lacustis* |
| *Phacus hamatus* | *Oscillatoria chlorina* |
| *Phacus helicoides* | *Peridiniopsis kevei* |
| *Phacus lismorensis* | *Peridinium pusillum* |
| *Phacus pyrum* | *Phacus pyrum* |
| *Phormidium aerugineo-coeruleum* | *Pseudoanabaena* sp . |
| *Planktosphaeria gelotinosa* | *Scenedesmus abundans* |
| *Pseudoanabaena* sp. | *Scenedesmus acuminatus* |
| *Rhabdogloea smithii* | *Scenedesmus brasiliensis* |
| *Scenedesmus acuminatus* | *Scenedesmus denticulatus* |
| *Scenedesmus denticulatus* | *Scenedesmus platydiscus* |
| *Scenedesmus platydiscus* | *Scenedesmus quadricanda* |
| *Scenedesmus producto-capitatus* | *Staurastrum planctonicum* |
| *Scenedesmus quadricauda* | *Staurastrum sexangulare* |
| *Scenedesmus wuhanensis* | *Staurodesmus aristiferus* |
| *Staurastrum dilatatum* | *Surirella capronii* |
| *Staurastrum planctonicum* | *Surirella linearis* |
| *Staurastrum sexangulare* | *Surirella robusta* |
| *Staurastrum tetracerum* | *Synedra acus* |
| *Staurodesmus aristiferus* | *Synedra* sp. |
| *Sttombomonas fluviatilis* | *Tetrastrum elegans* |
| *Surirella robusta* | *Tetrastrum glabrum* |
| *Synedra acus* | *Trachelomonas hispida* |
| *Synedra* sp. | *Trachelomonas scabra* |
| *Tetraedron trigonum* | *Trachelomonas volvocina* |
| *Trachelomonas lismorensis* | - |
| *Trachelomonas nodsoni* | - |
| *Trachelomonas oblonga* | - |
| *Trachelomonas similis* | - |
| *Trachelomonas volvocina* | - |
| *Treubaria crassispina* | - |
| *Trochiscia reticuraris* | - |

Table S2 Zooplankton species in the Lianhe Reservoir during thermal stratification and mixing periods in 2017.

| The thermal stratification period | The mixing period |
| --- | --- |
| Rotifera | Rotifera |
| *Keratella cochlearis* | *Collotheca sp.* |
| *Monostyla lunaris* | *Conochilus unicornis* |
| *Polyarthra trigla* | *Filinia maior* |
| *Trichocerca capucina* | *Keratella cochlearis* |
| Cladocera | *Lecane eutarsa* |
| *Bosmina longirostris* | *Polyarthra trigla* |
| *Diaphanosoma birgei* | *Synchaeta pectinata* |
| *Moina micrura* | Cladocera |
| Copepoda | *Bosmina longirostris* |
| *Mesocyclops leuckarti* | *Bosminopsis deitersi* |
| *Neodiaptomus Schmackeri* | *Ceriodaphnia cornuta* |
| - | Copepoda |
| - | *Mesocyclops leuckarti* |
| - | Neodiaptomus schmackeri |
| - | *Thermocyclops hyalinus* |
